# Unraveling the role of lipid metabolism in ALS risk: a Mendelian randomization analysis using GWAS data

**DOI:** 10.1186/s13023-026-04503-2

**Published:** 2026-07-20

**Authors:** Houwen Zhang, Chunrong Li, Jialin Yu, Yu Liang, You Wu, Fangzheng Cao

**Affiliations:** 1 Department of Neurology, The Second Affiliated Hospital of Zhejiang Chinese Medical University (Xinhua Hospital of Zhejiang Province), Hangzhou, 310005 China; 2 The Second Clinical Medical College of Zhejiang Chinese Medical University, Hangzhou, China; 3 Center for Rehabilitation Medicine, Department of Neurology, Zhejiang Provincial People’s Hospital (Affiliated People’s Hospital, Hangzhou Medical College), Hangzhou, Zhejiang 310014 China

**Keywords:** Amyotrophic lateral sclerosis (ALS), Lipid traits, Mendelian randomization (MR), Diacylglycerol, Triacylglycerol

## Abstract

**Background:**

Amyotrophic lateral sclerosis (ALS) is a progressive neurodegenerative disease. Lipid metabolism is closely related to neuronal function and energy homeostasis, but the genetic association between specific lipid species and ALS risk remains unclear.

**Objective:**

This study aimed to investigate the potential causal associations between genetically predicted lipid species and ALS risk using a two-sample Mendelian randomization (MR) approach.

**Methods:**

Summary-level GWAS data for 179 lipid species were obtained from 7,174 Finnish participants in the GeneRISK cohort. ALS GWAS data included 29,612 ALS cases and 122,656 controls. The inverse variance weighted (IVW) method was used as the primary MR approach, supplemented by MR-Egger, weighted median, weighted mode, and simple mode analyses. False discovery rate (FDR) correction was applied across all lipid traits based on IVW P values. Sensitivity analyses were conducted to assess heterogeneity, horizontal pleiotropy, and robustness.

**Results:**

After FDR correction, genetically predicted higher levels of diacylglycerol (DAG) (18:1_18:1), phosphatidylcholine (PC) (16:1_18:1), PC (18:0_18:1), phosphatidylethanolamine (PE) (O-16:1_18:2), and several triacylglycerol (TAG) species were associated with increased ALS risk. Phosphatidylinositol (PI) (16:0_18:1) showed only a nominal protective association and did not remain significant after FDR correction. Sensitivity analyses did not indicate substantial heterogeneity or horizontal pleiotropy.

**Conclusion:**

This MR study provides genetic evidence supporting potential associations between specific lipid species and ALS risk. These findings highlight lipid metabolism as a relevant pathway in ALS susceptibility.

**Supplementary Information:**

The online version contains supplementary material available at https://doi.org/10.1186/s13023-026-04503-2.

## Introduction

ALS is a progressive neurodegenerative disorder characterized by degeneration of upper and lower motor neurons, leading to muscle weakness, paralysis, and ultimately death [1]. The disease is clinically and genetically heterogeneous, and its etiology remains incompletely understood [2]. Genetic susceptibility [3], environmental exposure, and metabolic disturbance have all been implicated in ALS risk [4, 5]. Among these factors, lipid metabolism is particularly relevant because it is essential for neuronal function, energy homeostasis, and membrane integrity.

Some research indicates that ALS patients often exhibit elevated serum lipid and cholesterol concentrations [6–8]. However, a comprehensive meta-analysis has yet to confirm a definitive association between ALS clinical symptoms and plasma lipid levels [9]. Furthermore, the role of lipid metabolism in ALS progression and prognosis remains contentious. Certain studies suggest that higher plasma cholesterol levels and hypertriglyceridemia may confer neuroprotective effects and are associated with longer survival times [7, 10–12]. In contrast, other investigations propose that the accumulation of cholesterol and its metabolites can induce oxidative stress in motor neurons, potentially increasing the risk of ALS development [13–15]. Additionally, after adjusting for confounding factors, the association of hypertriglyceridemia as a potential protective factor for ALS became non-significant [8, 16]. Nonetheless, prior studies have demonstrated significant variability in how lipid profiles correlate with different clinical phenotypes of ALS [17]. The conclusions largely stem from observational research, which can be influenced by confounding variables, thus restricting the capacity to make definitive causal conclusions.

Mendelian randomization (MR) uses genetic variants as instrumental variables (IVs) to estimate the potential causal effect of an exposure on an outcome [18]. Because genetic variants are randomly allocated at conception and remain stable throughout life, MR can reduce confounding and reverse causation compared with conventional observational studies [19].

This study applied two-sample MR to investigate the potential association between genetically predicted lipid traits and ALS risk, with the aim of identifying lipid species that may warrant further mechanistic investigation.

## Methods

**Fig. 1:**
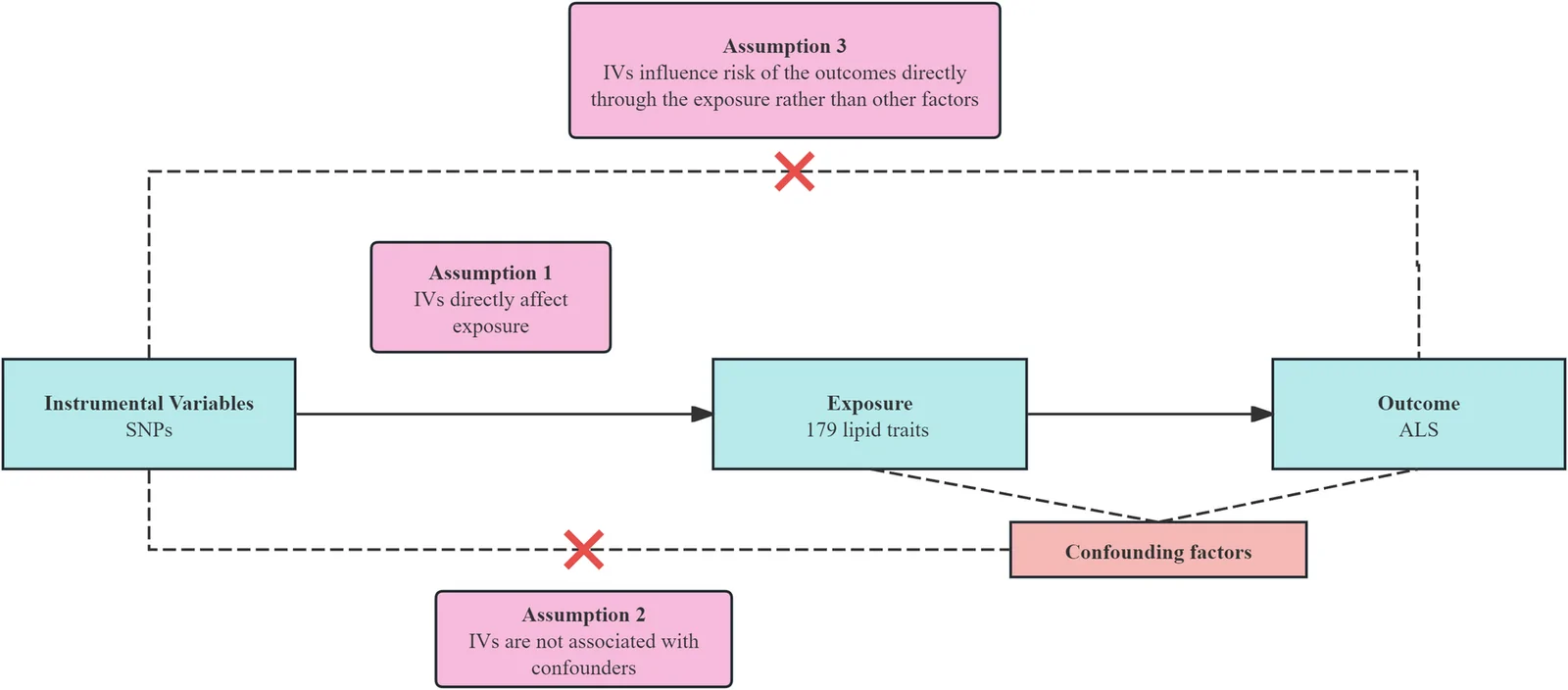
Schematic representation of the MR framework used to explore the potential association between lipid traits and ALS. The diagram illustrates the key assumptions of MR: Assumption 1, where IVs, represented by SNPs, directly affect lipid exposure; Assumption 2, where IVs are not associated with confounding factors; and Assumption 3, where IVs influence ALS risk solely through lipid exposure and not through other pathways

Figure 1 provides a visual representation of the study design, offering a comprehensive overview.

### Data sources

The lipid data for this study were derived from a large-scale genome-wide association study (GWAS) involving 7,174 Finnish individuals from the GeneRISK cohort [20]. This cohort was assessed using shotgun lipidomics to measure 179 lipid species. The study’s high-throughput lipidomic measurements enabled a detailed exploration of the genetic underpinnings of lipid metabolism.

The ALS summary statistics used in this study were obtained from a large cross-ancestry GWAS, which was predominantly composed of individuals of European ancestry [21]. This study included 29,612 ALS cases and 122,656 control subjects, representing one of the most comprehensive genetic investigations of ALS to date. The GWAS integrated individual-level genotype data from 117 cohorts categorized according to genotyping platforms and analyzed approximately 10.4 million genetic variants, providing a broad assessment of the genetic architecture of ALS.

### IVs selection

To determine an appropriate threshold for instrumental-variable selection, we first evaluated the availability of genome-wide significant single nucleotide polymorphisms (SNPs) for the 179 lipid species using the conventional threshold of *P* < 5 × 10^− 8^. Under this threshold, only 25 lipid species had two or more independent SNPs, indicating that most lipid traits would have insufficient instruments for MR analysis. Therefore, we adopted a relatively relaxed threshold of *P* < 1 × 10^− 5^ for the primary analysis to preserve lipidome-wide coverage and maintain comparability across lipid species [22].

To ensure the independence of the selected instruments, SNPs were clumped using an LD threshold of R² < 0.001 within a 10,000-kb window [23]. SNPs with a minor allele frequency ≤ 0.01 were excluded. During harmonization with the ALS GWAS, variants unavailable in the outcome dataset and ambiguous palindromic SNPs were removed. To reduce the risk of weak instrument bias, the F statistic was calculated for each retained SNP–lipid instrument, and only instruments with F statistics > 10 were included in the MR analysis [24].

### MR analysis

Five MR methods were utilized to explore the potential association between lipid traits and ALS risk: inverse variance weighted (IVW) [25], MR-Egger [26], weighted median, weighted mode, and simple mode [27]. The primary method used was IVW, as it offers the most reliable estimates when there is no horizontal pleiotropy. The results were expressed as odds ratios (OR) with 95% confidence intervals (CI). To account for multiple testing, false discovery rate (FDR) correction was applied across all lipid traits based on the IVW P values. Associations with FDR-adjusted P values < 0.05 were considered statistically significant after multiple-testing correction, whereas associations with raw IVW P values < 0.05 but FDR-adjusted P values ≥ 0.05 were considered nominal associations [28].

### Sensitivity analysis

Several sensitivity analyses were performed to assess the robustness and validity of the MR estimates. First, Cochran’s Q test was used to evaluate heterogeneity of SNP effects [29]. To explore potential horizontal pleiotropy, we applied the MR-Egger intercept test [30] and the MR-PRESSO global test, which assess whether genetic variants influence the outcome through pathways other than the exposure of interest [31]. Additionally, a leave-one-out analysis was conducted, systematically removing each SNP to check for consistency [32]. To further evaluate potential horizontal pleiotropy, SNP-trait annotation was performed for instrumental SNPs of lipid species showing significant or suggestive associations using the LDtrait module of LDlink. LDtrait was used to examine whether the instrumental SNPs, or their proxy variants in linkage disequilibrium, had previously been associated with ALS, motor neuron disease, or major non-lipid traits potentially related to ALS risk. The European reference population was used for LD estimation, and proxy variants were considered at an R^2^ threshold of ≥ 0.8.

## Results

### IVs selection

Using the instrument-selection strategy described above, 4,529 independent SNP–lipid instruments were initially selected across the 179 lipid species. After harmonization with the ALS GWAS, 3,662 SNP–lipid instruments were retained for the final MR analyses. All retained instruments had F statistics greater than 10, suggesting that weak instrument bias was unlikely to materially affect the MR estimates. Detailed information on the selected and harmonized instruments is provided in Supplementary Tables S1 and S2.

### MR analyses of lipid traits on ALS

**Fig. 2:**
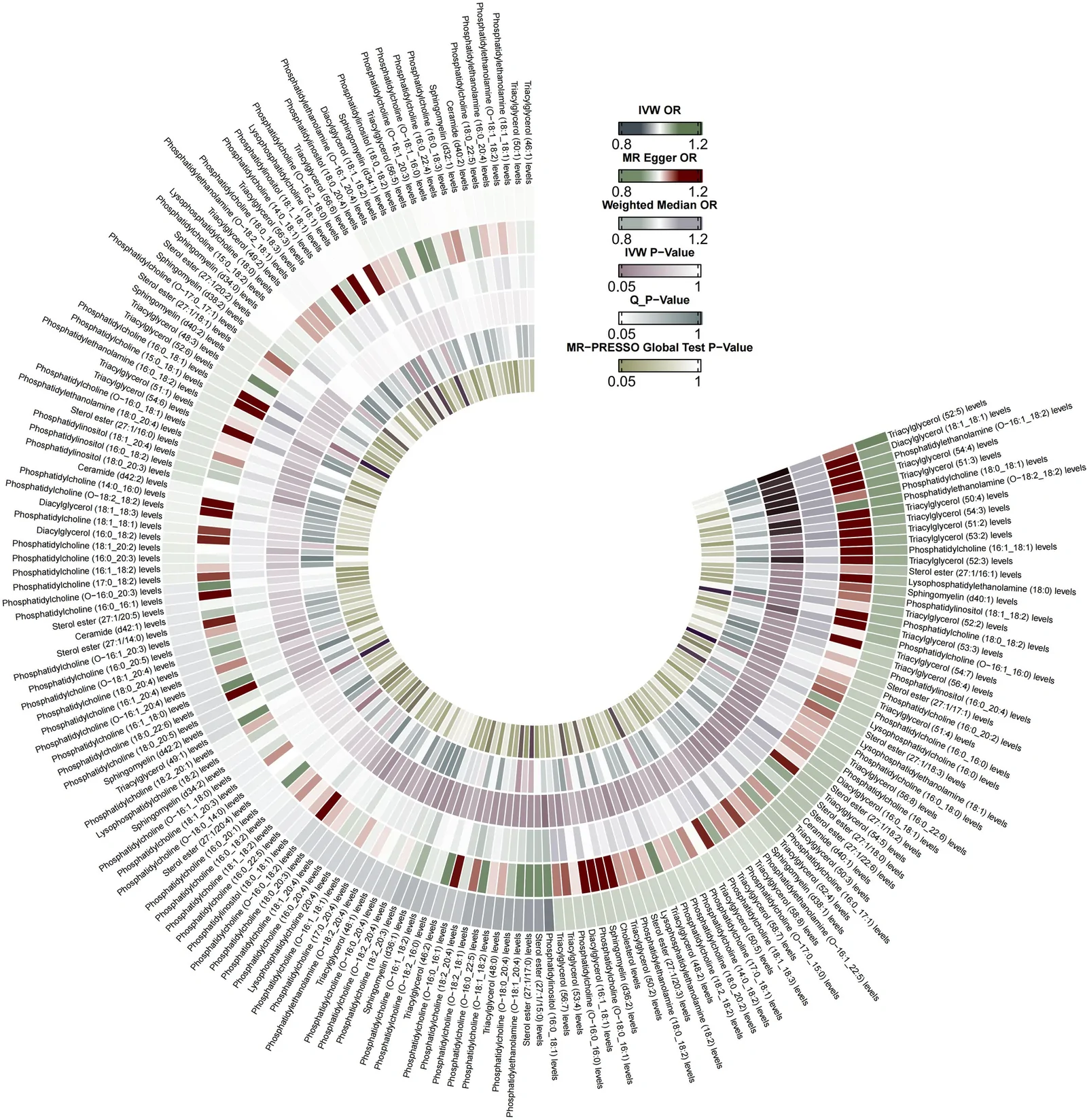
Circular visualization of MR results. Each segment represents a specific lipid species, with color gradients indicating effect sizes and statistical significance across different MR methods, including inverse variance weighted (IVW), MR-Egger, and weighted median approaches. The inner rings display OR, while the outer rings depict P values from IVW, Cochran’s Q test, and MR-PRESSO global test

**Fig. 3:**
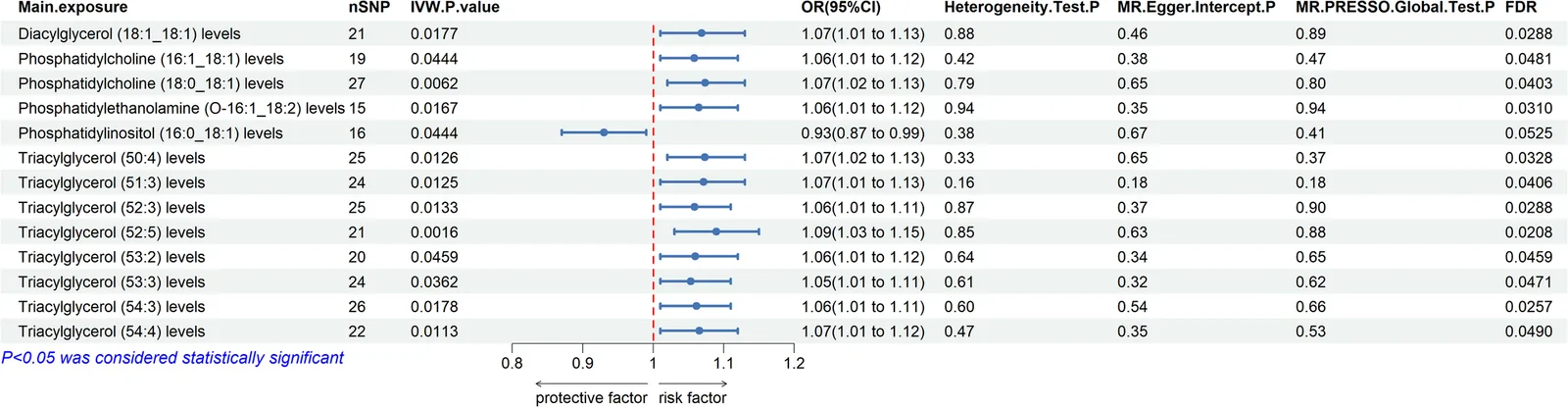
Forest plot summarizing the MR associations between lipid traits and ALS risk. The forest plot includes lipid species, number of SNPs, inverse variance weighted (IVW) P values, OR with 95% confidence intervals, heterogeneity test P values, MR-Egger intercept P values, and MR-PRESSO global test P values. The FDR-adjusted P values are also provided. The forest plot visually represents the ORs, where values > 1 suggest an increased risk, and values < 1 suggest a protective effect. The dashed red line at OR = 1 indicates no effect. FDR-adjusted P values < 0.05 were considered statistically significant after multiple-testing correction, whereas raw IVW P values < 0.05 but FDR-adjusted P values ≥ 0.05 were considered nominal associations

**Fig. 4:**
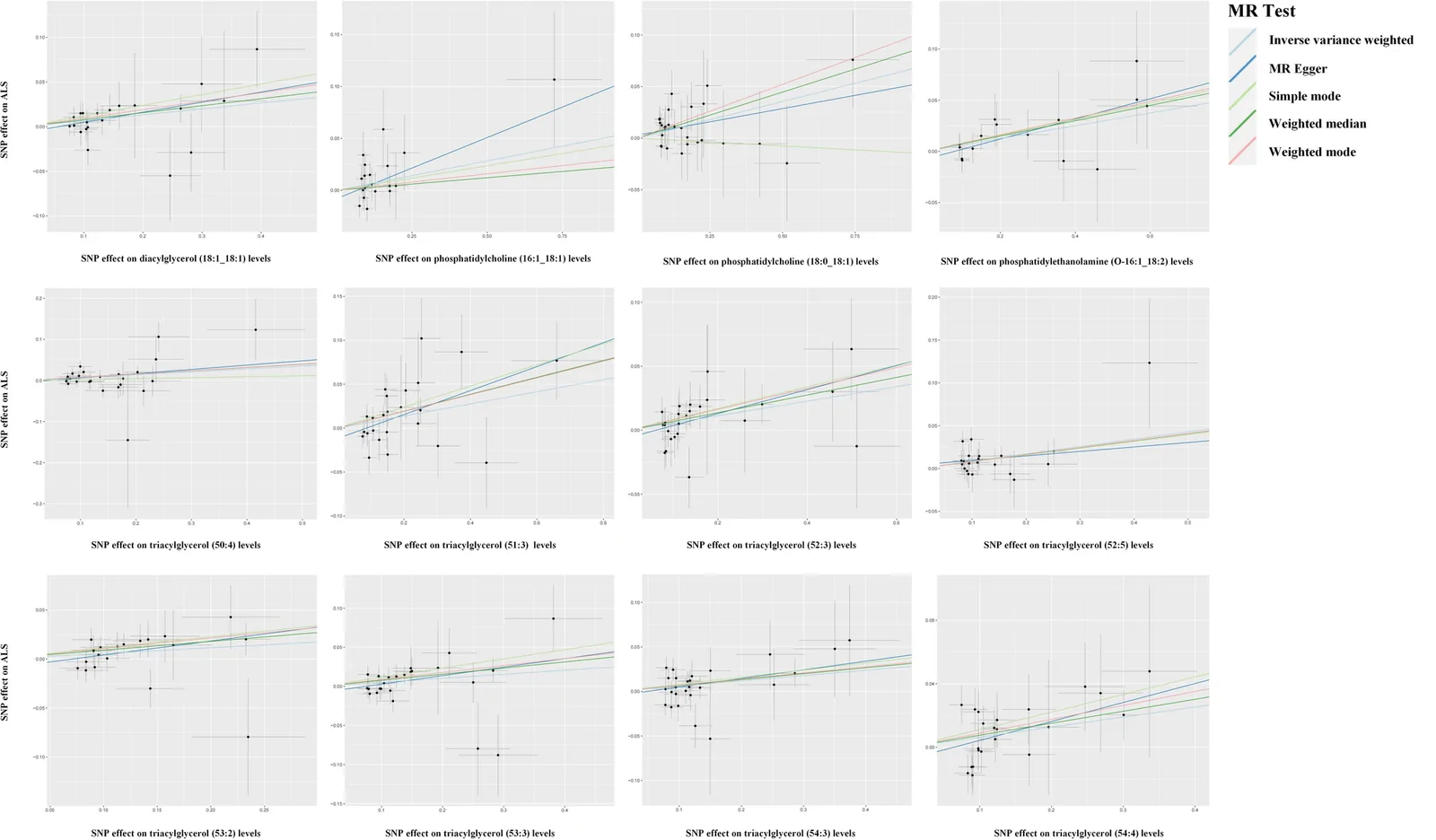
Scatterplot of the MR analysis. The associations between SNPs and lipid traits are presented alongside individual SNP associations with ALS, marked by black dots. Vertical and horizontal lines indicate the 95% confidence interval of the OR for each SNP. The slope of these lines reflects the MR estimate derived from the MR methods

The overall associations between genetically predicted lipid traits and ALS risk are presented in Fig. 2, with detailed MR estimates provided in Supplementary Table S3. Lipid species showing nominal or FDR-significant evidence of association in the IVW analysis are summarized in Figs. 3 and 4.

After FDR correction across all lipid traits, genetically predicted higher levels of DAG (18:1_18:1), PC (16:1_18:1), PC (18:0_18:1), PE (O-16:1_18:2), and several TAG species, including TAG (50:4), TAG (51:3), TAG (52:3), TAG (52:5), TAG (53:2), TAG (53:3), TAG (54:3), and TAG (54:4), remained significantly associated with an increased risk of ALS. In contrast, PI (16:0_18:1) showed only a nominal protective association with ALS risk and did not remain significant after FDR correction.

### Sensitivity analyses

**Fig. 5:**
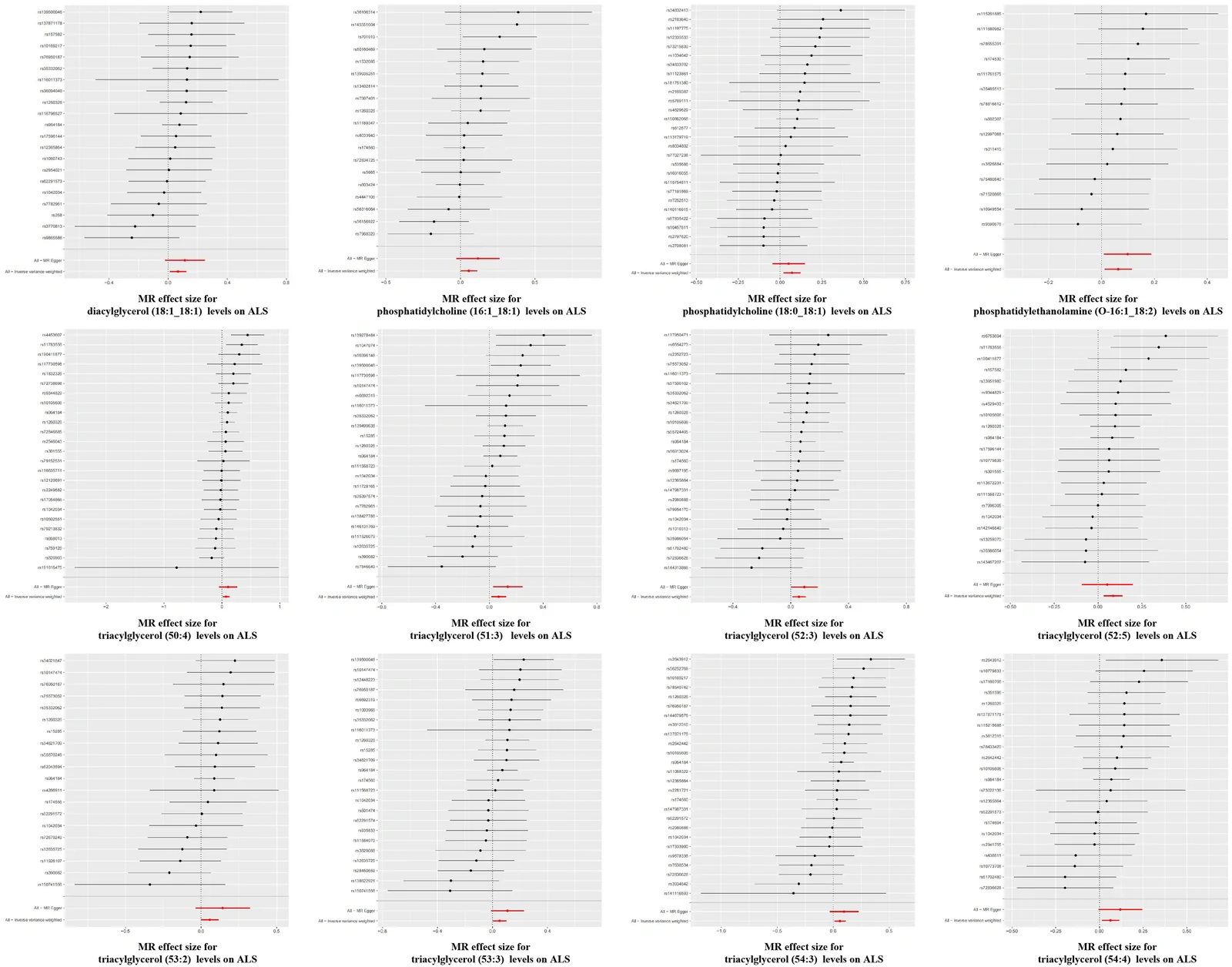
Forest plot for MR analysis. This plot shows the SNP-specific effects of lipid-associated variants on ALS risk. The solid black horizontal lines depict the effect estimates for each SNP in relation to ALS risk, calculated using the Wald ratio method. Each black line is linked to a particular SNP identified in lipid-related GWAS. Conversely, the red line indicates the overall effect estimate of lipid-associated SNPs on the risk of ALS, which is obtained by aggregating the effects from all individual SNPs

**Fig. 6:**
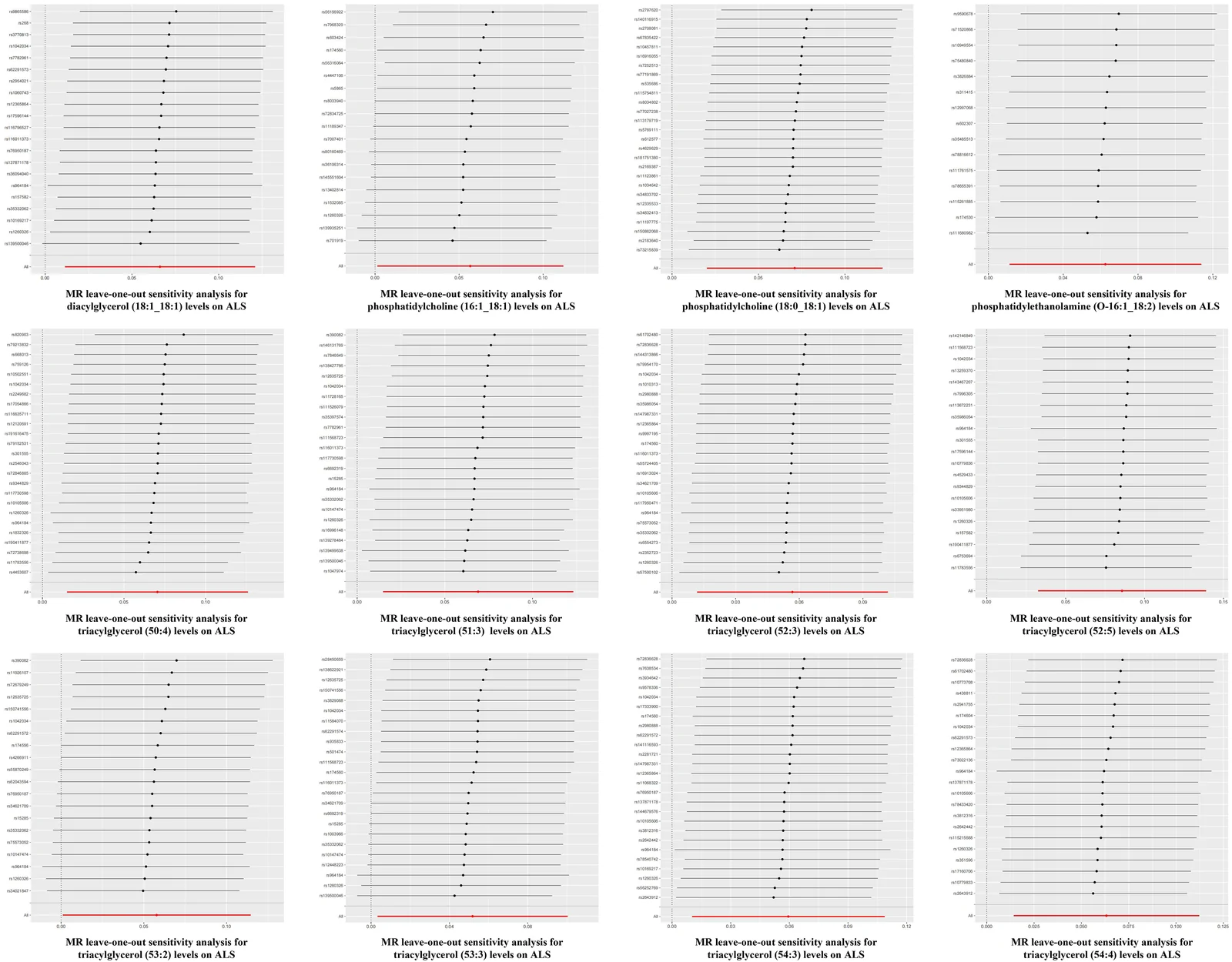
Plot illustrating the leave-one-out analysis for MR associations between lipid species showing statistical evidence of association and ALS risk. By systematically omitting each individual SNP, the effect of the remaining SNPs in the meta-analysis is recalibrated to detect variations in the outcomes that occur after the exclusion of each SNP

Sensitivity analyses supported the robustness of the main MR findings. Cochran’s Q test showed no substantial heterogeneity among the selected SNPs (*P* > 0.05; Supplementary Table S4). MR-Egger intercept and MR-PRESSO analyses provided no evidence of directional horizontal pleiotropy or outlier variants (*P* > 0.05; Fig. 5 and Supplementary Tables S5 and S6). Leave-one-out analysis indicated that the MR estimates were not driven by any single SNP (Fig. 6). LDtrait-based SNP–trait annotation further showed no evident associations between the instrumental SNPs, or their LD proxy variants, and ALS, motor neuron disease, or major non-lipid traits potentially related to ALS risk.

## Discussion

In this study, we used two-sample MR to investigate the potential associations between genetically predicted lipid species and ALS risk. Several lipid species, including DAG (18:1_18:1), PC (16:1_18:1), PC (18:0_18:1), PE (O-16:1_18:2), and several TAG species, showed genetic evidence of association with increased ALS risk. These findings suggest that specific lipid species may be involved in ALS susceptibility.

Through metabolomics analysis, DAG was identified as a significantly altered subpathway within lipid metabolism [33]. One study reported a negative association between DAGs and pre-symptomatic ALS compared to healthy participants [34]. However, our findings provided genetic evidence that a specific DAG species, DAG (18:1_18:1), may be associated with ALS risk. This is consistent with previous lipidomic studies reporting dysregulation of DAG-related pathways in ALS [35]. Among various DAG species, DAG (18:1_18:1) exhibited the highest loading score and significance in orthogonal partial least squares discriminant analysis, indicating a robust association with neurofilament light chain [35]. Additionally, DAG (18:1_18:1) is made up of a pair of oleic acid chains (18:1). Importantly, increased levels of oleic acid have been noted in patients with ALS [36], which may contribute as substrates for the production of proinflammatory mediators that play a role in the pathogenesis of ALS [37]. Furthermore, oleic acid exerts proinflammatory effects by generating reactive oxygen species (ROS) and activating the phosphorylation of mitogen-activated protein kinase (MAPK) and/or protein kinase C (PKC) [38, 39]. DAG is a bioactive signaling lipid involved in processes such as cytoskeleton regulation, neuronal development, inflammation, immune cell signaling, and apoptosis [40, 41], all of which are closely linked to ALS pathology. Together, these findings suggest that DAG (18:1_18:1) may represent a candidate lipid species for further investigation in ALS-related lipid dysregulation.

Phospholipids are essential for the metabolism of the nervous system and are associated with the development of neurodegenerative disorders, including Alzheimer’s disease, Parkinson’s disease, and multiple sclerosis [42, 43]. In patients with ALS and corresponding animal models, there is a notable increase in PC concentrations [44]. This rise in PC levels may promote the activity of phospholipase A2, which subsequently leads to the release of lipid mediators such as eicosanoids, potentially initiating inflammatory responses in ALS [44]. The release of arachidonic acid has been correlated with the production of prostaglandins and lipid peroxidation [45]. Our MR analysis provided genetic evidence supporting a potential association between higher levels of certain PC species and ALS risk, complementing previous observational findings. Additionally, PE (O-16:1_18:2) showed genetic evidence of association with increased ALS risk. However, given the limited evidence regarding the role of PE species in ALS, further studies are needed to clarify their biological relevance.

Research indicates that individuals with ALS exhibit a greater occurrence of hypertriglyceridemia, and there is a positive correlation between triglyceride levels and life expectancy [8, 16]. However, in larger sample studies, after adjusting for confounding factors, this association was not observed [46–48]. Nonetheless, other research has reported that ALS patients exhibit increased clearance of peripheral triglycerides [49]. Moreover, Biosigner analysis identified certain long-chain triglycerides as predictors of disease progression, with lower levels associated with better prognoses [44]. The differences observed in these results might be linked to a higher number of confounding variables and the influence of reverse causality. By utilizing genetic variants as IVs, MR analysis can help reduce these biases when estimating potential causal relationships between exposures and outcomes [19]. Our results suggested that specific very long-chain TAGs may be associated with increased ALS risk. These associations may be related to the involvement of long-chain fatty acids in cellular stress and protein aggregation pathways implicated in ALS, although the underlying mechanisms require further experimental validation [50].

Nevertheless, several limitations should be acknowledged. First, although two-sample MR can reduce confounding and reverse causation, it relies on the validity of the instrumental variables. We used a relatively relaxed threshold of *P* < 1 × 10^− 5^ for instrumental-variable selection to preserve lipidome-wide coverage. Although all retained instruments had F statistics > 10, residual weak instrument bias cannot be completely excluded. Second, horizontal pleiotropy remains a potential concern. MR-Egger intercept, MR-PRESSO, leave-one-out analysis, and LDtrait-based SNP–trait annotation did not indicate obvious pleiotropy, but these approaches cannot fully exclude unknown or unreported pleiotropic pathways. Third, the lipid GWAS was conducted in Finnish individuals, whereas the ALS GWAS was based on a cross-ancestry meta-analysis. Although the ALS GWAS was predominantly composed of individuals of European ancestry, residual bias from population stratification and ancestry-specific LD patterns cannot be fully excluded. Finally, MR provides genetic evidence for potential associations but does not directly establish biological mechanisms or therapeutic targets. Further experimental and clinical studies are needed to clarify how these lipid species may contribute to ALS susceptibility.

## Conclusion

In summary, this two-sample MR study provides genetic evidence supporting potential associations between specific lipid species and ALS risk. Genetically predicted higher levels of DAG (18:1_18:1), PC (16:1_18:1), PC (18:0_18:1), PE (O-16:1_18:2), and several TAG species were associated with an increased risk of ALS. These findings suggest that lipid metabolism may be involved in ALS susceptibility and identify candidate lipid species for future mechanistic investigation. Further experimental and clinical studies are needed to clarify the biological mechanisms underlying these associations.

## Supplementary Information

Below is the link to the electronic supplementary material.

Supplementary Material 1

## Data Availability

The summary statistics for the lipid GWAS were obtained from the GWAS Catalog hosted by the European Bioinformatics Institute, specifically between the accession numbers GCST90277238 and GCST90277416. Data on ALS used in our research was acquired from the OpenGWAS database of the Medical Research Council-Integrative Epidemiology Unit (MRC-IEU) available at https://gwas.mrcieu.ac.uk/. Further details can be located in the original GWAS studies cited in our manuscript.
